# Effectiveness of distributing pocket cards in improving the behavior, attitude, and knowledge regarding proper medication use among junior high school students in Japan

**DOI:** 10.3389/fpubh.2023.1296073

**Published:** 2024-01-11

**Authors:** Chihiro Sakai, Kazuhiro Iguchi, Tomoya Tachi, Yoshihiro Noguchi, Aki Hisamatsu, Shingo Katsuno, Hitomi Teramachi

**Affiliations:** ^1^Laboratory of Community Pharmacy, Department of Pharmacy, Gifu Pharmaceutical University, Gifu, Japan; ^2^Laboratory of Hospital Pharmacy, Department of Pharmaceutical Sciences, Nagoya City University, Nagoya, Japan; ^3^Laboratory of Clinical Pharmacy, Department of Pharmacy, Gifu Pharmaceutical University, Gifu, Japan; ^4^Education Center of Green Pharmaceutical Sciences, Gifu Pharmaceutical University, Gifu, Japan; ^5^Gifu Pharmaceutical University, Gifu, Japan

**Keywords:** self-medication, education, junior high school, school-based intervention, Japan

## Abstract

**Objective:**

This study aimed to explore the effectiveness of distributing pocket cards with summaries of key information on appropriate medication usage after the implementation of a structured school-based medication education program for junior high school students in Japan.

**Methods:**

A total of 227 3rd-grade high school students participated in the intervention. Students who received the program without the provision of pocket cards in 2022 were included in the comparison group, and students who took the program with the provision of pocket cards in 2023 were included in the intervention group. After propensity score matching, the final sample of *N* = 116 comprised *n* = 58 comparison group participants and *n* = 58 intervention group participants. Questionnaires were administered at baseline, end-of-class, and 3-month follow-up to assess the changes in behavior, attitude, and knowledge scores.

**Results:**

The matched intervention group showed significantly lower scores at the 3-month follow-up than the matched comparison group. The results of the multiple linear regression analysis showed that for both groups, only the attitude scores were significantly correlated with the behavior scores. In addition, regardless of the baseline scores, the matched intervention group demonstrated smaller or negative changes in scores at the 3-month follow-up.

**Conclusion:**

Overall, the results of this study did not support the effectiveness of distributing pocket cards after in-class intervention. However, the usefulness of medication education intervention was confirmed. These results emphasize the need to explore other supplemental teaching tools to further enhance the impact of structured medication education programs.

## 1 Introduction

According to the World Health Organization, self-medication, as an element of self-care, is defined as “the selection and use of medicines (including herbal and traditional products) by individuals to treat self-recognized illnesses or symptoms” (1). In recent years, medication literacy has gained global attention as a key factor in proper medication use-associated behavior (2, 3). For example, among adolescents, junior high school students with lower medication literacy are significantly more likely to engage in inappropriate self-medication practices (4). In addition, lower medication literacy has been associated with longer-term usage of medications such as painkillers and antacids (5).

Previous studies have shown that adolescents begin to self-administer medications at junior high school age. For instance, in a survey conducted in Canada, 75.9% of 651 junior high school students (including students from the 7th, 8th, and 9th grades) reported that they had taken medication independently (6). In Japan, among 348 3rd-graders from five public junior high schools, 32.3% of the male and 33.7% of the female students reported that they had taken medication without speaking to an adult, and the rates increased to 37.1 and 42.2% for the male and female students, respectively, of a total of 1,420 first-graders at seven public high schools (7). Therefore, improving the medication literacy is necessary, particularly among adolescents.

In Japan, which is one of the world’s oldest societies, a variety of national-level cost-containment measures have been implemented in response to the increase in national medical expenses. One of the primary measures is the promotion of self-medication. Specifically, the government introduced a new over-the-counter medication retail system and self-medication tax deduction in 2006 and 2017, respectively, to advance the use of over-the-counter medications for non-severe symptoms with the intention of reducing patients’ hospital visits (8, 9). In response to the need for acquiring the knowledge and skills to administer safe self-medication because of the expanding use of over-the-counter medication, Japan’s Ministry of Education, Culture, Sports, Science and Technology revised the national education guidelines for junior high school and high school students, adding new content to teach the proper use of medicines in the health and physical education fields (10). In accordance with the revision of the national education guidelines, all junior high school students aged 14 or 15 and all high school students aged 17 or 18 were required to acquire basic knowledge of medication and self-medication, including the role of medication in treatment, dose–response relationship, and importance of following drug fact labels.

Considering all these contexts, the authors of this study conducted multiple surveys using both regional- and national-level samples to collect information on the behavior, attitude, and knowledge regarding medication use among elementary, junior high, and high school students in Japan (11, 12). Based on the results of these studies, the authors developed a structured school-based medication education program aimed at promoting students’ behavioral and attitudinal changes as well as improving their basic medication literacy in collaboration with physical education teachers and school nurses (13). However, the results of a large-scale cross-sectional study conducted by the authors revealed the possibility of insufficient effectiveness of classes provided at schools attended by survey participants (14). Another study conducted by the authors, in which Bayesian network analysis for causal inference was adopted, suggested that an improvement in knowledge of appropriate medicine use might lead to the acquisition of favorable attitudes, which could result in positive behavioral changes (14).

Based on the findings of the previous studies (11, 13), the authors developed a school-based medication education program for junior high school students. The program has been provided to junior high school students, and its effectiveness in changing participant behaviors, attitudes, and knowledge has been confirmed (15). Therefore, the present study examined whether distributing pocket cards with basic information on proper medication use after carrying out a medication education program would further promote behavioral and attitudinal changes and knowledge acquisition. To investigate this, the authors compared a group of students who were provided only the program with another group of students who were provided both the program and pocket cards.

The present study examined the following three hypotheses:

*H1*: Compared to the students not provided with pocket cards, the students provided with pocket cards show higher behavior, attitude, terminology, and understanding scores at the 3-month follow-up.

*H2*: The effect of scores on terminology and understanding on the behavior score at the 3-month follow-up is greater among the students provided with pocket cards than among the students not provided with pocket cards.

*H3*: Regardless of the behavior, attitude, terminology, and understanding scores at baseline, the students provided with pocket cards show a greater increase in scores at the 3-month follow-up than those of the students not provided with pocket cards.

## 2 Methods

### 2.1 Participants and setting

The 50-min medication education program developed by the authors was delivered to all 3rd-grade students aged 14 or 15 in a public junior high school in Seki City in 2022 and 2023. The pocket cards with the key points of the program were provided to the students who received the program in 2023, and they were asked to carry the cards with them. The group of students who received the program in 2022 without pocket card provision was enrolled as the comparison group, whereas the group of students who received the program in 2023 with pocket card provision was enrolled as the intervention group.

The contents of the program were structured to align with the Course of Study for Junior High School Students (10), and the following contents were taught in the class: the role of natural healing power and medication; classification of medication, including the difference between prescribed and over-the-counter medication; rules for medication use, including dosage and administration; how to read labels of over-the-counter medication; dose–response relationship and mechanism of how medications work in the body. To facilitate students’ understanding, a variety of visual materials and experimental demonstrations were presented in class.

To evaluate the changes in students’ behavior, attitude, and knowledge, three types of in-person anonymous surveys were administered by homeroom teachers in classrooms to all the participants who were included in the program. The surveys were conducted at baseline, end-of-class, and 3-month follow-up. In total, 114 and 94 students responded to the surveys in 2022 and 2023, respectively, among which the numbers of valid responses were 113 and 94 (99.1 and 100.0%), respectively.

### 2.2 Instruments

The questions asked in the survey had been used in our previous studies (11, 12, 14) and had been assessed by school teachers to determine whether the terms used were understandable enough for junior high school students. The term “medication” was clearly defined and indicated at the beginning of the survey as follows: “Please tell me what you think about medication. ‘Medication’ used in this questionnaire refers to the medication you are given at the hospital or buy at a community pharmacy or a drug store. It includes not only medication for internal use but also compresses, external medicines, and disinfectants used for injuries and other occasions. It also includes household medication, eye drops, troches, and inhalants. However, it does not include nutritional supplements or energy drinks.”

The questionnaire comprised 13 single-and multiple-choice questions. Questions regarding general healthcare and medication use included the following: (1) What do you do when you are in poor physical condition? (i.e., go to sleep early, take medicine at home, consult with families, consult with a teacher, see a doctor, consult a pharmacy, other); (2) For what purpose do you use medication? (i.e., stomachache, headache, cold, fever, toothache, allergies, car sickness, other); (3) Who do you consult when you use medication? (i.e., parents/grandparents, brothers/sisters, friend, doctor/dentist, pharmacist, schoolteacher, I have medication that I take regularly, there is no medicine I take regularly, other); and (4) Have you ever done the following: purchased medication on your own judgment, received medication from a friend, gave medication to a friend? Questions regarding behavior, attitude towards, and knowledge of medication use included the following: (1) When you use medication, what kinds of things are you careful about? (i.e., read the description, check the dosage, check the dosage time, check that I had a meal, take medication with water, ensure the medication is suitable to my constitution, I do not care, other); (2) When you use medication, what do you think is important to be careful of? (i.e., read the description, check the dosage, check the dosage time, check that I had a meal, take medication with water, ensure the medication is suitable to my constitution, other); (3) What terminology do you know? (i.e., over-the-counter medicine, prescribed medicine, generic medicine, family pharmacy, medication notebooks, doping, and school pharmacist); and (4) Which items related to a medicine’s proper use do you know? (i.e., do not take medication with milk or juice; do not bite tablets or disassemble capsules; between meals is not the same as during meals; take medication for the indicated number of days; most medication has some side effects; do not overdose even if the medication does not work soon; do not double the dosage, even if you forget to take it once; cold over-the-counter medicine is symptomatic treatment). The baseline and 3-month follow-up surveys included questions on behavior, attitude, and knowledge, and the end-of-class survey included only questions regarding attitude. The respondents were asked to select “yes” for all choices that applied to them on each list.

### 2.3 Statistical analysis

Questions regarding the behaviors, attitudes, terminology, and knowledge of proper medication use were scored for each item, and the total scores for each domain were calculated, with the answer “Yes” counting as one point (14). According to this calculation method, the behavior scores ranged from 0 to 6. Similarly, the attitude, terminology, and knowledge scores ranged from 0 to 6, 0 to 7, and 0 to 8, respectively.

As the groups with and without the provision of pocket cards differed in group-level characteristics, propensity score matching was used to ensure that the intervention and comparison groups were as similar as possible. Propensity score matching is a method used to adjust for selection bias in non-randomized studies of causal effects (16). It is designed to improve the match between individuals in the intervention group and those in the comparison group using demographic or other characteristics. In this study, a propensity score for the participating students was created based on sex and total scores for behavior, attitude, and knowledge at baseline. For each intervention group participant, one control participant with the closest propensity score was selected as the matched participant.

Different statistical methods were used to test each hypothesis. For Hypothesis 1, an independent sample *t*-test was used to compare the scores on the four domains between the two groups. For Hypothesis 2, multiple linear regression was adopted to assess the strength of the relationship between the behavior and the variables that could affect it, namely attitude, terminology, and understanding, at the 3-month follow-up. For Hypothesis 3, the students in both the intervention and comparison groups were divided into two groups, namely students with lower scores at baseline and those with higher scores at baseline, utilizing the mean scores of each domain as cut-off scores. Then, the difference in scores on the four domains between the baseline and 3-month follow-up surveys (the score in the 3-month survey subtracted from the score in the pre-survey) was calculated for each participant in each group. For within-subgroup comparisons, differences between the intervention and comparison groups were tested. For between-subgroup comparisons, differences between the subgroups were tested. All analyses were performed using SPSS version 27.

## 3 Results

After nearest-neighbor propensity score matching, the final sample of *N* = 116 comprised *n* = 58 comparison participants and *n* = 58 intervention participants; unmatched participants were excluded from the analysis. The results suggested that propensity score matching reduced the differences in the percentages of male and female students as well as the differences in the baseline scores on the four domains between the matched comparison and matched intervention groups (Table 1).

**Table 1:** Participant characteristics and scores at baseline before and after propensity score matching.

	Comparison group		Intervention group	
	Unmatched (*n* = 113)	Matched (*n* = 58)	Unmatched (*n* = 94)	Matched (*n* = 58)
Demographic				
Female (%)	44.7	50.0	56.1	50.0
Scores at baseline (score (SD))				
Behavior	3.16	3.33	3.76	3.47
Attitude	3.70	3.98	4.75	4.16
Terminology	4.31	4.69	4.89	4.67
Understanding	4.34	5.34	6.27	5.31

### 3.1 Test of hypothesis 1

Compared to the 58 participants included in the matched comparison group, the 58 participants included in the matched intervention group demonstrated significantly lower behavior, attitude, and understanding scores at the 3-month follow-up (behavior: *t* (109) = 2.927, *p* = 0.004; attitude: *t* (109) = 2.286, *p* = 0.024; understanding: *t* (107) = 2.439, *p* = 0.016) (Table 2).

**Table 2:** Comparison of scores in post- and 3-month surveys.

	Matched comparison group (*n* = 58)	Matched intervention group (n = 58)		
	M of score (SD)	M of score (SD)	Cohen’s d and 95% C.I.	*t*-test
Post-test				
Attitude	5.10 (1.25)	4.77 (1.49)	0.24 (−0.18, 0.84)	1.293
3-month follow-up				
Behavior	4.38 (1.28)	3.64 (1.37)	0.56 (0.24, 1.24)	2.927**
Attitude	5.21 (1.31)	4.60 (1.47)	0.43 (0.080, 1.13)	2.286*
Terminology	5.31 (1.52)	5.23 (1.64)	0.053 (−0.51, 0.68)	0.279
Understanding	6.68 (1.98)	5.77 (1.93)	0.47 (0.17, 1.66)	2.434*

* and ** indicate significance at the 95 and 99% levels, respectively.

### 3.2 Test of hypothesis 2

In Models 1 and 2, multiple linear regressions were fitted to explain the scores of changes in behavior based on the scores of changes in attitude, terminology, and understanding. Overall, Models 1 and 2 explained 47.2 and 46.6% of the variations, respectively, and were significantly useful in explaining the behavior score at the 3-month follow-up (Model 1: *F* (3, 49) = 14.604, *p* < 0.001; Model 2: *F* (3, 52) = 15.148, *p* < 0.001).

For Models 1 and 2, with a one-unit increase in the attitude scores, the behavior scores increased by 0.519 and 0.590, respectively, and these changes were significant (Model 1: *t* (49) =5.085, *p* < 0.001; Model 2: *t* (52) =4.460, *p* < 0.001) (Table 3). With a one-unit increase in the terminology scores, the behavior scores for Models 1 and 2 increased by 0.113 and 0.140, respectively; however, these changes were not significant (Model 1: *t* (49) = 0.113, *p* = 0.294; Model 2: *t* (52) =1.026, *p* = 0.310). With a one-unit increase in the understanding scores, the behavior scores for Models 1 and 2 increased by 0.028 and 0.148, respectively; these changes were also not significant (Model 1: *t* (49) = 0.396, *p* = 0.694; Model 2: *t* (52) =1.089, *p* = 0.281).

**Table 3:** Multiple-linear regression models predicting the behavior scores at the 3-month follow-up.

	Model 1 Matched comparison group	Model 2 Matched intervention group
Constant	0.837	−0.746
Attitude	0.519**	0.590**
Terminology	0.113	0.140
Understanding	0.028	0.148
R square	0.472	0.466

* and ** indicate significance at the 95 and 99% levels, respectively.

Model 1: F (3, 49) = 14.604, *p* < 0.001.

Model 2: F (3, 52) = 15.148, *p* < 0.001.

### 3.3 Test of hypothesis 3

For the within-subgroup analysis of participants with lower behavior, attitude, terminology, and understanding scores at baseline, the matched intervention group showed smaller changes in scores than those of the matched comparison group, and changes in the behavior and attitude scores were statistically significant (behavior: *t* (49) = 2.240, *p* = 0.030; attitude: *t* (60) = 2.268, *p* = 0.013) (Table 4). On the other hand, among the students whose behavior, attitude, terminology, and understanding scores at baseline were higher than average, the matched intervention group showed negative changes, and the behavior and understanding scores at the 3-month follow-up were lower than those at baseline, with statistical significance for behavior and understanding (behavior: *t* (58) = 2.958, *p* = 0.004; understanding: *t* (54) = 2.203, *p* = 0.032).

**Table 4:** Subgroup analysis of the degree of changes in scores between the baseline and 3-month follow-up.

		Students with lower scores at baseline				Students with higher scores at baseline				(In the matched comparison group)^a^		(In the matched intervention group)^b^	
		Matched comparison group	Matched intervention group	Within-subgroup		Matched comparison group	Matched intervention group	Within-subgroup		Between-subgroup		Between-subgroup	
	*M* of scores at baseline	M (SD) (*n*)	M (SD) (*n*)	Cohen’s d (95% C.I.)	*t*-test	M (SD) (*n*)	M (SD) (*n*)	Cohen’s d 95% C.I.	*t*-test	Cohen’s d 95% C.I.	*t*-test	Cohen’s d 95% C.I.	*t*-test
Behavior	3.40	1.68 (1.36) (*n* = 28)	0.87 (1.18) (*n* = 23)	0.63 (0.083, 1.53)	2.24*	0.47 (0.94) (*n* = 30)	−0.40 (1.30) (*n* = 30)	0.76 (0.28, 1.45)	2.96**	1.04 (0.60, 1.82)	3.97**	1.02 (0.57, 1.97)	3.66**
Attitude	4.07	2.12 (1.67) (*n* = 34)	1.25 (1.27) (*n* = 28)	0.58 (0.10, 1.63)	2.27*	−0.042 (1.20) (*n* = 24)	−0.52 (1.64) (*n* = 25)	0.33 (−0.35, 1.31)	1.16	1.44 (1.36, 2.96)	5.43**	1.22 (0.97, 2.57)	4.43**
Terminology	4.68	1.29 (1.68) (*n* = 24)	0.87 (1.22) (*n* = 23)	0.29 (−0.44, 1.29)	0.98	,15 (1.31) (*n* = 34)	0.30 (1.49) (*n* = 30)	−0.11 (−0.85, 0.55)	−0.44	0.78 (0.36, 1.93)	2.92**	0.41 (−0.20, 1.34)	1.49
Understanding	5.33	2.04 (2.39) (*n* = 25)	1.14 (1.82) (*n* = 28)	0.43 (−0.27, 2.06)	1.55	0.78 (1.58) (*n* = 32)	−0.21 (1.77) (*n* = 24)	0.60 (0.089, 1.89)	2.20*	0.64 (0.20, 2.31)	2.39*	0.75 (0.35, 2.35)	2.70**

a Comparison between students with lower scores and those with higher scores at baseline in the matched comparison group.

b Comparison between students with lower scores and those higher scores at baseline in the matched intervention group.

For the between-subgroup analysis, in the matched comparison group, the students with lower scores at baseline showed greater positive changes in their scores for all four domains (behavior: *t* (56) = 3.970, *p* < 0.001; attitude: *t* (56) = 5.432, *p* < 0.001; terminology: *t* (56) = 2.918, *p* = 0.005; understanding: *t* (55) = 2.388, *p* = 0.020). Similarly, in the matched intervention group, the students with lower scores at baseline had greater positive improvements in the scores for all four domains, and changes in the behavior, attitude, and understanding scores were statistically significant (behavior: *t* (51) = 3.662, *p* < 0.001; attitude: *t* (51) = 4.431, *p* < 0.001; understanding: (50) = 2.704, *p* = 0.009).

## 4 Discussion

The present study examined the effectiveness of distributing pocket cards after providing a school-based medication education program developed by the authors, in comparison with providing the program alone, for improving the behavior, attitude, and knowledge regarding medication use among junior high school students.

Overall, the examination of the three hypotheses yielded unexpected results. While both the matched intervention and matched comparison groups showed an increase in the scores at the 3-month follow-up, which can be seen by comparing the results in Tables 1, 2, the results of testing Hypothesis 1 demonstrated that the matched intervention group had lower behavior, attitude, and understanding scores than those of the matched comparison group at the 3-month follow-up. The analysis of Hypothesis 2 revealed that only the attitude score had a significant effect on the behavior score, not only in the matched control group, but also in the matched intervention group. In our previous study utilizing Bayesian inference, we reported a causal relationship among the four domains in that acquiring the knowledge on approprate medication use leads to the acquisition of favorable attitudes, which may result in behavioral changes (14). The results of this study and the authors’ previous study (14) were consistent in terms of the implication that attitude could be the most influential factor affecting behavior. Therefore, attitude changes might be the key to promoting behavioral changes.

This study also posited that regardless of the scores for the four domains at baseline, pocket cards would be useful for all participants to achieve a substantial increase in scores at the 3-month follow-up, which was tested through Hypothesis 3. However, the within-subgroup comparison showed an overall smaller positive change in scores in the matched intervention group than in the matched comparison group. In addition, in the between-subgroup comparison, compared to the students with lower scores at baseline, the students with higher scores at baseline tended to show significantly smaller or negative changes, indicating a decline in the scores after the intervention. This may be explained by the fact that participants with relatively high baseline scores could easily reach the highest level and had difficulty demonstrating further improvement. In contrast, participants with relatively low baseline scores may have more room for improvement.

Given that the examination of all three hypotheses showed unexpected results, while both groups received exactly the same medication education class, it is possible that they were not similar, even though propensity score matching was carried out. This implied that matching based only on sex and scores for the four domains at baseline might have been insufficient and that other variables such as those regarding the participants’ other characteristics should have been included in the matching. This study adopted pocket cards as supplemental teaching material to further enhance the impact of the medication education program on the participants; however, the results of this study did not demonstrate their usefulness. Thus, while pocket cards are a relatively low-cost supplemental teaching material, the impact of pocket cards on behavioral and attitudinal changes remains controversial. On one hand, Shearer et al. (17) reported that distributing pocket cards or stickers contributed to promoting adult participants’ desired behavior, stressing their convenience and feasibility. On the other hand, another study reported the insufficiency of a simple traditional tool that included the provision of a virtual educational program and pocket cards to improve malnutrition or nutritional treatment awareness (18). Given that there are reports that support their effectiveness and ineffectiveness in promoting behavioral and attitudinal changes, it may be necessary to explore the factors attributing to these disparitiess. In addition, exploring other forms of supplemental educational material that could help reinforce the impact of in-class program may be important.

The limitations of this study include its focus on a single junior high school with a small sample size, limited demographic variables used in group matching, and inability to confirm the effectiveness of pocket cards. In particular, despite that it is generally uncommon in Japan to ask socio-economic status-related questions in surveys for children and adolescents and to use student academic performance-related variables in social science studies, appending the demographic factors that could have an association with medication use in propensity score matching may be of use in such a study. Nonetheless, this study is the first to examine whether pocket cards can be used as supplementary educational material for teaching proper medication use and enhancing medication literacy in a junior high school setting. Furthermore, the results of this study implied that the medication education program itself had a positive impact on increasing the scores, suggesting that it promotes favorable changes in behavior, attitude, and knowledge among junior high school students. Therefore, further exploration of evidence-based supplementary teaching tools with promising effects may be needed to increase the effectiveness of medication education programs.

## Data availability statement

The raw data supporting the conclusions of this article will be made available by the authors, without undue reservation.

## Ethics statement

The studies involving humans were approved by Gifu Pharmaceutical University. The studies were conducted in accordance with the local legislation and institutional requirements. The ethics committee/institutional review board waived the requirement of written informed consent for participation from the participants or the participants' legal guardians/next of kin because The purpose of the survey was explained to the principals of the participating schools, and written consent was obtained. At the beginning of the questionnaire, it was noted that the questionnaire was not a requirement, and a student could stop at any time. Completion of the questionnaire was treated as consent for participation.

## Author contributions

CS: Formal analysis, Writing – original draft. KI: Formal analysis, Writing – review & editing. TT: Formal analysis, Writing – review & editing. YN: Formal analysis, Writing – review & editing. AH: Writing – review & editing. SK: Writing – review & editing, Conceptualization, Data curation, Project administration. HT: Conceptualization, Data curation, Funding acquisition, Project administration, Supervision, Writing – review & editing.
