# Supplementary material for: Effectiveness of distributing pocket cards in improving the behavior, attitude, and knowledge regarding proper medication use among junior high school students in Japan
Source: Front Public Health. 2024 Jan 11;11:1296073. doi: 10.3389/fpubh.2023.1296073 (PMC10835787; doi:10.3389/fpubh.2023.1296073)
Supplement: Supplementary file 1 [file Image_1.pdf]

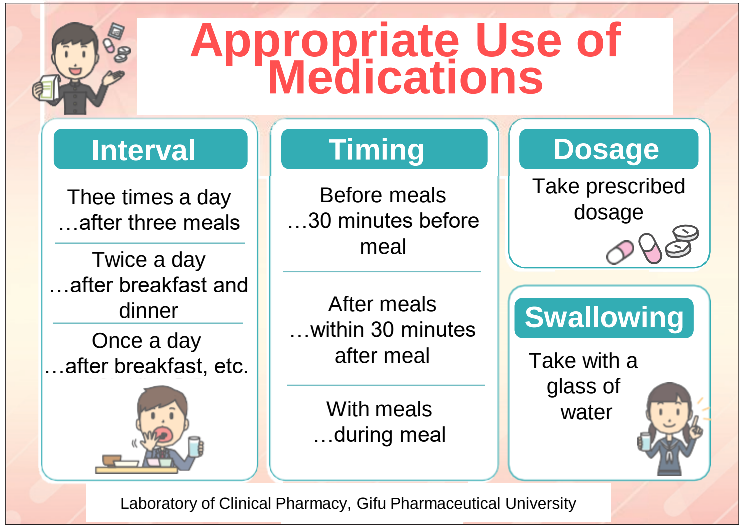

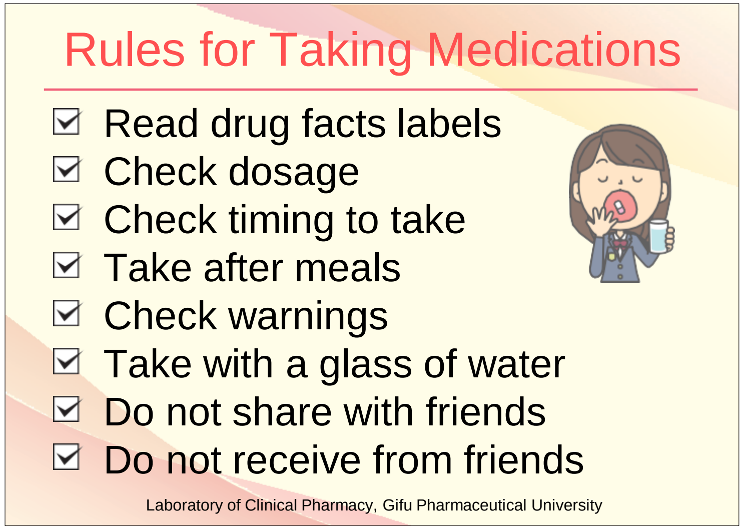

Note.
The text on this card is translated from Japanese language to English language for publication purposes.
The text and images on this card are owned by the corresponding author, and may not be reproduced without permission.
